# Inter-Individual Differences in Executive Functions Predict Multitasking Performance – Implications for the Central Attentional Bottleneck

**DOI:** 10.3389/fpsyg.2022.778966

**Published:** 2022-05-11

**Authors:** André J. Szameitat, Brunel Students, Caitlin Ball

**Affiliations:** Center for Cognitive Neuroscience (CCN), Division of Psychology, Department of Life Sciences, College of Health and Life Sciences, Brunel University London, Uxbridge, United Kingdom

**Keywords:** psychological refractory period (PRP), dual-task performance, multitasking, executive functions, individual differences, action control, passive queuing

## Abstract

Human multitasking suffers from a central attentional bottleneck preventing parallel performance of central mental operations, leading to profound deferments in task performance. While previous research assumed that the deferment is caused by a mere waiting time (refractory period), we show that the bottleneck requires executive functions (EF; active scheduling account) accounting for a profound part of the deferment. Three participant groups with EF impairments (dyslexics, highly neurotics, deprived smokers) showed worse multitasking performance than respective control groups. Three further groups with EF improvements (video-gamers, bilinguals, coffee consumers) showed improved multitasking. Finally, three groups performed a dual-task and different measures of EF (reading span, rotation span, symmetry span) and showed significant correlations between multitasking performance and working memory capacity. Demands on EF during multitasking may cause more errors, mental fatigue and stress, with parts of the population being considerably more prone to this.

## Introduction

**FIGURE 1:**
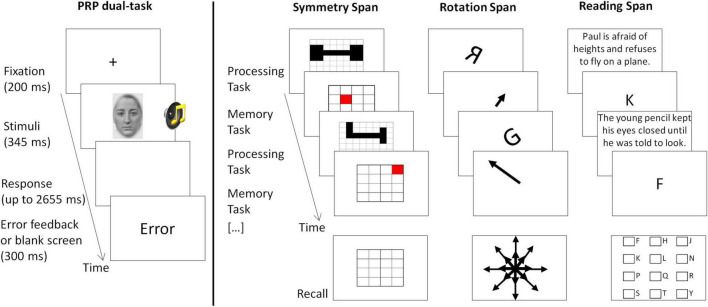
Illustration of the trial design of the PRP dual-task (studies 1 and 2) and the three complex working memory span tasks (study 2). In the PRP dual-task participants responded using both hands on a computer keyboard. As soon as the number of required responses was given, the response registration period was terminated. In some studies, the numbers “1” or “2” instead of male or female faces were used as visual stimuli. In the span tasks (Foster et al., 2015), responses were given using a computer mouse. In the processing task, each screen showing the task (depicted above) was followed by another screen registering the answer (not shown above, presenting a question such as “Are these shapes symmetrical?” with two answer boxes, “Yes” or “No,” to be clicked with the mouse).

Multitasking, i.e., the concurrent performance of two tasks, typically results in severe costs in form of prolonged response times (RTs) and increased error rates (Pashler, 1994). One source of such costs is a central attentional bottleneck preventing the parallel processing of certain mental operations. As a consequence, the tasks compete for the processing by the bottleneck, resulting in interference. In addition, the processing of one task has to wait until the other task has been processed by the bottleneck mechanism, the so-called refractory period (Telford, 1931; Broadbent, 1958). This bottleneck seems virtually immutable and is affecting a large number of mental processes central to human cognition, such as decision making or memory retrieval (Carrier and Pashler, 1995; Dux et al., 2006; Tombu et al., 2011). Even basic processes, such as pressing a button in response to a stimulus (speeded choice-response tasks) can defer a second response by half a second or more (paradigm of the Psychological Refractory Period, PRP, Figure 1; also see Supplementary Figure 2). When driving a car at 30 mph, this would increase the brake distance by approx. 7 m, illustrating the severity of the effect. While in popular models of bottleneck processing the refractory period is the sole source of multitasking costs, a further source has been suggested^1^.

This potential second source of multitasking costs is related to the wider implications the presence of a bottleneck might have on task processing. More specifically, it has been suggested that the interference between the tasks, which arises from the presence of the bottleneck, is resolved by executive functions (EF), such as the inhibition of one task while the other is processed and the switching of the bottleneck between the tasks (De Jong, 1995; Meyer and Kieras, 1997; Logan and Gordon, 2001; Szameitat et al., 2002, 2006; Luria and Meiran, 2003; Schubert, 2008; Wu and Liu, 2008; Koch et al., 2018). Demands on such EF might provide a further source of multitasking costs. In the context of the central attentional bottleneck, this theory has been termed the active-scheduling account. However, other authors suggested that EF are not needed to coordinate the processing of the tasks at the stage of the bottleneck (Jiang et al., 2004; Lehle et al., 2009; Hartley et al., 2012). Instead, the processing would work purely on a first-come first-served basis, a theory which has been called the passive queuing account.

A number of studies have found evidence supporting the active-scheduling account. One of the most commonly used tasks to assess bottleneck processing is the PRP task, in which participants are presented with two stimuli, S1 and S2, which both require a speeded choice-response, R1 and R2 (with their corresponding response times RT1 and RT2). S1 and S2 are either presented with a short temporal offset (stimulus onset asynchrony, SOA) or simultaneously (SOA 0 ms). Participants usually have to respond to the stimuli in a certain order, which implies that the bottleneck has to process the tasks in this order as well. Evidence for active scheduling was provided, for example, by Hirsch et al. (2017) who showed that the task pairs constituting a PRP dual-task are organized on a hierarchically higher level, which seems inconsistent with a plain passive queuing account (see also De Jong, 1995). Support for such higher-level coordination has also been provided by Strobach et al. (2021b) who showed that the time it takes to adjust the processing order of the component tasks depends on the nature of the preceding trial. Strobach et al. (2021a) also showed that participants can actively prepare a specific processing order of the tasks (cf. also De Jong, 1995; Luria and Meiran, 2003), again a finding inconsistent with a mere passive first-come first-serve approach. Somewhat indirect evidence for active scheduling in dual-tasks suffering from a bottleneck has been provided by Hirsch et al. (2018) who showed that the amount of multitasking costs in a PRP task correlates with task switching costs. Importantly, task-switching costs have been linked to cognitive control processes managing the task sets (Monsell, 2003). Finally, it has been suggested that only dual-task practice, but not single-task practice, trains EF, such as switching operations, which even transfer to modified dual-tasks (Liepelt et al., 2011; Strobach, 2020).

The active scheduling approach is also supported by neuroimaging data (Marois and Ivanoff, 2005). For example, several studies employing functional magnetic resonance imaging (fMRI) have shown that performance of a PRP dual-task activates lateral-prefrontal cortices (lPFC) over-additively more than predicted by the sum of the component tasks (Saylik et al., in press; Szameitat et al., 2002; Schubert and Szameitat, 2003; Stelzel et al., 2009), and that the lPFC is stronger activated if the order in which the participants have to respond to the task switches from one trial to the next as compared to when it stays constant (Szameitat et al., 2006; Stelzel et al., 2008). Furthermore, Stelzel et al. (2008) were able to demonstrate that the lPFC areas activated by dual-task coordination can be differentiated from the lPFC areas linked to the maintenance of the task-set [see also Strobach et al. (2021a) for a similar conclusion based on behavioral findings]. To complement the findings from fMRI studies, Kübler et al. (2019) have shown that disrupting the function of the lPFC by means of transcranial magnetic stimulation (TMS) also disrupts specifically the coordination of the processing order of the tasks. The fact that the lPFC areas activated by PRP dual-tasks are generally associated with EFs and task-coordination is in support of the active scheduling account.

However, the alternative assumption of the passive queuing account, which states that the tasks are simply processed on a first-come first-served basis without any additional coordination, has also received empirical support. One key support for the first-come first-served approach comes from studies which show that the times at which the stimuli arrive at the bottleneck determine the order in which the bottleneck processes them (Sigman and Dehaene, 2006; Hendrich et al., 2012; Strobach et al., 2018). However, the finding that such central arrival times can determine or affect processing order does not rule out that under different task conditions the processing order may be determined by hierarchically higher control processes (De Jong, 1995; Luria and Meiran, 2003; Leonhard et al., 2011; Strobach et al., 2021a) and that EFs are required for other aspects besides task-order coordination, such as inhibition of the second task to not interfere with the first task or switching the bottleneck between tasks. Further evidence for the passive queuing account comes from the observation that increasing overlap of the tasks (i.e., shorter SOA), which can be postulated to increase the demands on EFs, does not result in the expected electrodermal responses (Hartley et al., 2012). Finally, in an fMRI study Jiang et al. (2004) were unable to observe any brain activation linked to dual-task processing, again supporting the passive queuing approach.

The current study aimed at providing further evidence to resolve the controversy between active scheduling and passive queuing approaches by testing whether inter-individual differences in EF capabilities affect multitasking performance.

## Experiment Series 1

We aimed to resolve the debate about passive queuing vs. active scheduling by assessing the multitasking performance of populations of participants who are known to have either impaired (dyslexics; highly neurotics; nicotine deprived smokers) or improved (video-gamers; bilinguals; coffee consumers) EF capabilities as compared to respective control groups (non-dyslexics; low neurotics; non-deprived smokers; non-gamers; monolinguals; caffeine abstinence). If multitasking indeed demands EF (active scheduling), the multitasking performance of these groups should also be impaired or improved, respectively. Conversely, if EF are not relevant to multitasking (passive queuing), then all groups should show comparable multitasking performance. To control for generically different performance levels of the experimental and control groups, we compared the groups with respect to multitasking *costs*, i.e., the relative slowing in the multitasking as compared to the single-task performance. The findings will help to resolve the long-standing debate whether the presence of a central attentional bottleneck demands additional EF or not. They will also allow a first insight into the question whether the involvement of such additional EF cost time, i.e., contribute to the deferment of the second response. Finally, the findings will allow insights into how person characteristics, i.e., inter-individual differences in executive function capabilities, affect the performance in multitasking situations suffering from a central attentional bottleneck.

### Overview of the Groups

We compared six groups of participants with known modulations, i.e., impairments or improvements, of their executive function capabilities with controls. Notably, the manipulations in executive function capabilities were caused by a variety of factors, such as child development (dyslexia), personality (neuroticism), substance withdrawal (nicotine deprivation), training (video gaming experience), plasticity (bilingualism), and substance use (caffeine). While for a single group it is conceivable to explain potential effects on multitasking performance by alternative factors besides executive functions, we argue that executive functions are the only common factor across all these six highly different groups (but also see sections “Experiment Series 2” and “General Discussion,” which address this point as well).

In the following we will discuss evidence about the above proposed modulation of executive function capabilities in more detail. While the key deficits in **dyslexia**, a neurodevelopmental disorder affecting roughly 5% of the population, are reading problems, it has been suggested that dyslexics also show impairments in EF which contribute to the reading problems (Brosnan et al., 2002). For example, Brosnan et al. (2002) showed deficits in inhibition and sequencing not only in children, but also in university students. Smith-Spark and Fisk (2007) have further shown that dyslexic adults perform poorer than non-dyslexic controls in complex working memory span tasks, which are a good indicator of EF capabilities (cf. Experimental Series 2 below). In a recent meta-analysis of 26 studies, Lonergan et al. (2019) found strong evidence for profound deficits in inhibition, switching and auditory working memory in children suffering from dyslexia. Taken together, there is strong evidence that dyslexia results in a deficit of EF. In the present study, we compared dyslexic participants, who self-reported having received an official diagnosis, with non-dyslexic participants (see Supplementary Information for a more detailed characterization of all participant groups).

High levels of **neuroticism** have been associated with experiencing more stress and anxiousness (Eysenck and Eysenck, 1986), impairing cognitive performance (Osorio et al., 2003). In particular, impairments have been suggested in more difficult tasks demanding executive functions (Studer-Luethi et al., 2012). For example, Szameitat et al. (2016) showed that high neuroticism is associated with increased multitasking costs, which was accompanied by lower activation of EF-related areas in the lPFC. This is in line with the finding that lesions to the lateral-prefrontal cortex impair EF and at the same time increase the risk to develop symptoms of high neuroticism (Forbes et al., 2014). Saylik et al. (2018) also showed that high neurotics were impaired only in tasks demanding switching and/or inhibition, but not in a task demanding the visuo-spatial sketchpad. Therefore, we conclude that high levels of neuroticism are associated with lower EF capabilities. In the present study, we compared extreme groups of high neurotics (mean Eysenck Personality Questionnaire score 18, range 16–24) with low neurotics (mean score 3.89, range 0–6).

Nicotine, typically consumed by smoking cigarettes or e-cigarettes, has been shown to improve cognitive performance in a variety of cognitive domains, including working memory, in smokers as well as non-smokers (Heishman et al., 2010). **Nicotine abstinence** in smokers, on the other hand, produces withdrawal symptoms which include the impairment of cognitive functions (Ashare et al., 2014; and nicotine consumption alleviates these impairments again: Atzori et al., 2008). As for dyslexia and high neuroticism, also the effects of nicotine deprivation appear to particularly affect EF, such as inhibition, working memory, and task switching (Dawkins et al., 2007; Harrison et al., 2009; Jansari et al., 2013), although the effects may not always be fully consistent (Butler and Le Foll, 2019). Therefore, we conclude that, overall, there is good evidence that nicotine deprivation impairs EF. In the present study, we compared nicotine deprived smokers (regular smokers who abstained for at least 50 min; self-rated nicotine deprivation 8.5 on a scale of 1–10) with smokers who had a cigarette directly before the session (mean deprivation score 1.9).

After having discussed three groups of participants who are likely to show impaired EF, we now discuss three groups which have been proposed to show improved EF. Frequent playing of **videogames** has been suggested to improve cognitive functions [Granic et al., 2014; see Boot et al., 2011 for a more critical discussion]. Besides effects on visual attention (Green and Bavelier, 2003), also positive effects on EF have consistently been demonstrated (Stanmore et al., 2017). For example, in the study by Colzato et al. (2010) video-gamers were better at task-switching [see Karle et al. (2010), Colzato et al. (2013) for examples that not all EF benefit in the same way]. Strobach et al. (2012) suggested that video-gamers (and non-gamers after practice) outperform non-gamers in dual-task and task-switching paradigms and suggested that videogaming results in improved executive control skills relevant for multitasking. These observations are in line with a meta-analysis showing improvements in inhibition, top-down attention, and multitasking/task-switching (Bediou et al., 2018). Interestingly, improvements of EF can be seen in all age groups, including preschool children (Yang et al., 2020) and the oldest old (McCord et al., 2020). In conclusion, it seems very well established that video-gaming experience improves executive function abilities. In the present study, we compared frequent video-gamers (on average 3.5 h/day action games, plus 1.7 h/d other games) with occasional or non-gamers (0.6 h/d action games, plus 0.7 h/d other games).

**Coffee** is known to have a generally stimulating effect, including improvement of cognitive functions (Cappelletti et al., 2015; McLellan et al., 2016). Consumption of caffeine has been shown to improve EF in tasks such as the Tower of London task (Killgore et al., 2014), grammatical and logical reasoning (Kohler et al., 2006; Kamimori et al., 2015), the Jansari Assessment of Executive Functions (Soar et al., 2016), and the Attention Network Test (Brunyé et al., 2010). However, the evidence is slightly mixed, with some studies failing to find effects on EF (e.g., Gottselig et al., 2006), which may be caused by the profound variety in study design (e.g., whether sleep-deprivation was induced), caffeine-dosage, and participant characteristics (e.g., whether they are regular caffeine users). We conclude that, overall, there is good evidence that coffee consumption has the ability to improve EFs. In the present study, we asked all participants to not consume any caffeinated drink for at least 4 h before the study and compared participants which then drank a cup of coffee (the experiment started approx. 30 min after coffee consumption) with participants who did not have any drink.

Bilingualism has been proposed to improve EF, mainly due to the constant use of EF to focus attention to the target language, to switch between languages, and to inhibit the currently non-used language (Bialystok et al., 2009). And while there is profound support for this suggestion (van den Noort et al., 2019; Grundy, 2020), there recently have also been doubts about the consistency of the finding that bilingualism benefits EFs (e.g., Lehtonen et al., 2018). The currently most likely solution to this debate might be that the concept of bilingualism itself and the potentially affected cognitive domains require a more fine-grained definition (DeLuca et al., 2019; Bialystok, 2021). Despite this debate, we included a study comparing bilingual with monolingual participants. Because we used a rather strict definition of bilingualism for the current study (the ability to speak at least two languages with the proficiency of a native tongue by the age of six) we had the assumption that the bilingual participants have improved EF as compared to monolingual participants (ignoring potential further languages learned later in life, e.g., in school).

Each group constituted an independent study with a separate control group, run by a different experimenter. Five of the six reported studies were final year undergraduate dissertation projects and one study was part of Ph.D. work. All studies were conducted at Brunel University London between 2013 and 2017. The data are based on 7 students’ projects and all projects were supervised by the first author, AS. All students worked independently and tested independent samples. Two studies are based on two combined datasets each, collected independently by different students. The studies were run by the following students: Dyslexia: CB; Smoking deprivation: JB, MB; Neuroticism: RS (Ph.D. student); Video-gaming: NG, AO; Bilingualism: LS; Caffeine: AS.

In the following the methods of all the different studies are described briefly, for more details see the Supplementary Information.

### Methods

#### Participants

All studies were individually approved by the Department of Life Sciences Ethics committee, Brunel University London, and were carried out in accordance with the Declaration of Helsinki. Participants gave written informed consent before participation and were debriefed after the study. Participants were either reimbursed with course credits, £10, or volunteered for free. Virtually all participants were students of Brunel University London between the age of 18 and 25.

In total 274 participants took part (Table 1). We excluded participants based on three criteria: (a) if they had more than 30% errors in any task condition, (b) if their data were more than 2.5 standard deviations from the respective group mean, and (c) if they didn’t fulfill the criteria for the group. Most participants were excluded in the nicotine deprivation study, because perceived nicotine cravings were too low after only 30–50 min of deprivation. All details on participant selection can be found in the Supplementary Information.

**TABLE 1:** Demographic information.

	Study					
	Dyslexia	Neuroticism	Nicotine deprivation	Video-gaming	Bi-lingualism	Coffee
**Group**	**Dyslexics**	**High neurotics**	**Deprived smokers**	**Frequent gamers**	**Bilinguals**	**Consumed coffee**

N Original	17	22	31	38	15	25
N Final	14	20	10	31	13	19
N females	7	10	6	10	8	14
N males	7	10	4	21	5	5
Age mean	22	21.362	20.333	19.895	22.333	19.75
Age range	20–28	18–27	18–25	18–26	19–28	18–23
Age s.d.	2.449	2.002	2.582	1.941	2.774	1.164

**Controls**	**Non-dyslexics**	**Low neurotics**	**Non-deprived smokers**	**Non-gamers**	**Mono-linguals**	**Caffeine abstinent > 4 h**

N Original	17	17	29	27	15	21
N Final	12	15	17	22	12	16
N females	8	8	10	13	5	12
N males	4	7	7	9	7	4
Age mean	21.9	23.501	21.917	21.727	20.727	22.125
Age range	20–31	18–26	19–29	18–27	18–23	18–27
Age s.d.	3.282	2.265	2.343	2.284	1.555	2.247

*N Original refers to participant number before selection, N Final to number after selection. Age data refer to the final sample and is given in years. s.d., standard deviation.*

#### Design

All studies were based on a mixed two-factorial design. Group is a between-subject factor with the levels experimental group (e.g., dyslexics, high neurotics, etc.) and control group (e.g., non-dyslexics, low neurotics, etc.). Task is a within subject factor with the levels single-task and dual-task (i.e., multitasking). The interaction between the two factors is of particular interest, because it reflects whether the decrements in dual-task performance as compared to single-task performance were different for the two groups. The dependent variables were response times (RT) for the single-tasks and for each of the two tasks in the dual-task (RT1 and RT2), as well as error rates (reflecting whether the trial as a whole was either correct, or whether one or more potential errors were made).

#### Materials and Procedure

We employed the dual-task paradigm of the psychological refractory period (PRP), which consisted of the combination of a visual and an auditory speeded choice response task (Figure 1, left panel). A trial in the visual single-task started with the presentation of a fixation cross for 200 ms, after which the visual stimulus was presented for 345 ms. The visual stimulus was either a male or female face (studies Dyslexia, Neuroticism, Smoking deprivation, Video-gaming, Bilingualism) or the numbers “1” or “2” (Coffee study) presented in the middle of the screen. Participants had to respond using their right hand by pressing the according buttons on a computer keyboard (“n” for male face/number 1; “m” for female/2, respectively). Participants had to respond within 2,655 ms after onset of stimulus presentation. After the response was given or 2,655 ms had passed since stimulus onset, either a blank screen or an error message was displayed for 300 ms, before the next trial started. A trial in the auditory task was identical except that instead of a visual stimulus, a blank screen was displayed and a sound was played *via* headphones for 345 ms. This sound was either a double-syllable (/haha/or/yaya/; studies Dyslexia, Neuroticism, Smoking deprivation, Video-gaming, Bilingualism) or a beep (300 Hz or 800 Hz; Coffee study). Participants responded using their left hand (“x” for/haha//300 Hz; “c” for/yaya//800 Hz). A trial in the dual-task was identical, except that both stimuli were presented at the same time (stimulus-onset-asynchrony, SOA, 0 ms) and participants had to respond to both stimuli using the same key mapping as in the single-tasks. Participants had to respond to the tasks in a given order (e.g., first to the auditory task) which was varied across blocks and specified by an instruction before each block. Participants received an error feedback if they pressed one or more wrong keys, and if they pressed the correct keys, but in the wrong order. In some studies, additional conditions were presented (e.g., SOA 1,000 ms, random task-order), which are not relevant to the current report.

Each condition (auditory single-task, visual single-task, dual-task (response order auditory- > visual), dual-task (visual- > auditory) was presented in two blocks of 35 trials each. For the analysis, the two single-tasks as well as the two dual-tasks were combined, resulting in 140 single-task and 140 dual-task trials. Before each condition, a self-paced instruction about the upcoming task was presented on the screen, which also served as the opportunity for a break. Order of conditions and trials were individually randomized. Before the main experiment, participants practiced all task conditions. A session lasted between 40 and 60 min. The task was presented using Presentation^2^.

### Results

The aim of these experiments was to assess whether groups of participants with impaired executive functions show impaired multitasking performance, and whether groups of participants with improved executive functions show improved multitasking performance. To control for potential generic differences between the groups, we calculated 2 × 2–factorial mixed ANOVAs for each study, with the factors Group [experimental group (e.g., dyslexics) vs control group (e.g., non-dyslexics)] and Task (single-task vs dual-task). Generally, the costs of multitasking are evident by poorer performance (increased RTs and error rates) in the dual-task as compared to the single-task. An executive function impairment/improvement specific to multitasking should be evident by a difference in these multitasking costs between the groups, which is reflected by the interaction term of the ANOVA.

**FIGURE 2:**
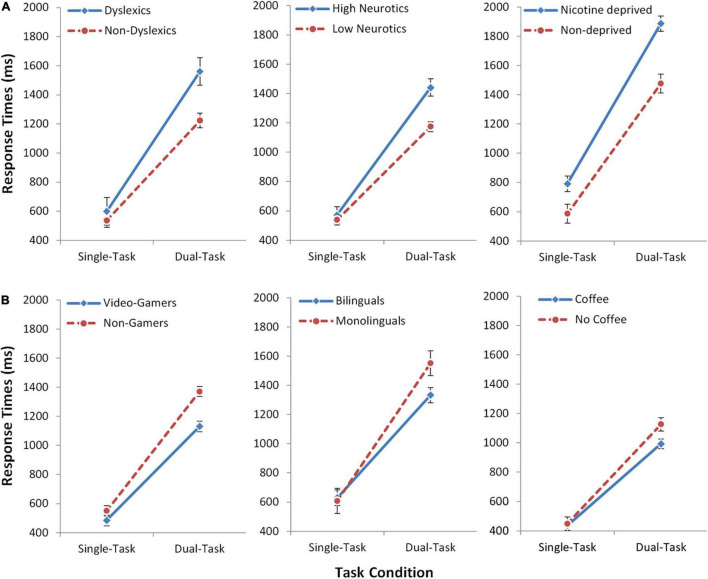
Experiment Series 1. Response times (ms) for the three independent groups of participants who are known to have impaired executive functions [upper panel, **(A)**] and for the three groups who are known to have improved executive functions [lower panel, **(B)**]. Dual-task costs are the relative slowing of response times in the dual-task (RT2) as compared to the single-task. Effects specific to multitasking are evident by increased or decreased dual-task costs, respectively, reflected in the interaction. Error bars show standard error of the mean (SEM).

First, we analyzed the response times. In the dual-task, we used the response times of the second task (RT2), because this is most sensitive to any form of dual-task costs (Pashler, 1994; see also Supplementary Information). In all six studies, participants generally showed profound multitasking costs of 797 ms (averaged across all studies and groups) and, consequently, the main effect of Task was significant in all studies (all *p* < 0.001). In most studies, there was also an overall difference between the groups, as reflected by a significant main effect of Group (all *p* < 0.05), except for the studies on bilingualism (*p* = 0.246) and caffeine (*p* = 0.066). Most importantly, in all six studies we observed a significant interaction between Group and Task in the expected direction (Figure 2 and Table 2; all *p*-values < 0.05). For the groups which are known to have impaired executive functions (dyslexics, highly neurotics, nicotine deprived), the multitasking costs were increased by 23–36% as compared to the respective control groups (Table 3). On the other hand, for the groups which are known to have improved executive functions (video-gamers, bilinguals, coffee consumers), the multitasking costs were lower by 20–25% as compared to the respective control groups. Additional analyses (see Supplementary Information) confirmed the interactions are not driven by a mere prolongation/shortening of the response selection stages in the experimental groups. This illustrates that individual differences in executive function capabilities affect multitasking performance. It also provides strong support for the active scheduling account of the central attentional bottleneck theory.

**TABLE 2:** Descriptive and inferential statistics of response times in Experiment Series 1.

	Study						
		Dyslexia	Neuroticism	Nicotine deprivation	Video-gaming	Bi-lingualism	Coffee
Sample Size—Group	N	14	20	10	31	13	19
Sample Size—Controls	N	12	15	17	22	12	16
**Descriptive statistics**							
RT1—Group	ms	1,137.987 ± 92.437	1,139.306 ± 59.602	1,404.892 ± 54.045	898.308 ± 41.727	1,062.903 ± 51.994	740.553 ± 30.329
RT1—Controls	ms	910.643 ± 53.490	943.242 ± 45.610	1,064.718 ± 71.431	1,082.978 ± 57.549	1,168.074 ± 81.499	849.682 ± 33.957
RT2—Group	ms	1,550.727 ± 108.602	1,441.061 ± 66.500	1,867.506 ± 74.244	1,130.659 ± 48.630	1,304.421 ± 64.617	972.926 ± 36.673
RT2—Controls	ms	1,223.956 ± 64.199	1,173.943 ± 38.914	1,478.106 ± 84.634	1,370.626 ± 61.550	1,504.094 ± 88.094	1,125.629 ± 55.805
Single-Task RT—Group	ms	616.588 ± 33.773	570.566 ± 16.439	781.345 ± 48.013	484.306 ± 17.165	620.496 ± 31.955	436.203 ± 15.961
Single-Task RT—Controls	ms	538.447 ± 29.271	537.876 ± 17.076	598.278 ± 34.196	549.808 ± 15.580	591.442 ± 26.602	448.435 ± 16.102
**Inferential statistics**							
Main effect Task		F(1, 24) = 253.237; ***p* < 0.001**; pη^2^.913	F(1, 33) = 437.802; ***p* < 0.001**; pη^2^ 0.93	F(1, 25) = 527.389; ***p* < 0.001**; pη^2^ 0.955	F(1, 51) = 578.369; ***p* < 0.001**; pη^2^ 0.919	F(1, 23) = 293.181; ***p* < 0.001**; pη^2^ 0.927	F(1, 33) = 584.044; ***p* < 0.001**; pη^2^ 0.947
Main effect Group		F(1, 24) = 6.135; ***p* = 0.021**; pη^2^ 0.198	F(1, 33) = 8.201; ***p* = 0.007**; pη^2^ 0.199	F(1, 25) = 9.871; ***p* = 0.004**; pη^2^ 0.283	F(1, 51) = 9.319; ***p* = 0.004**; pη^2^ 0.154	F(1, 23) = 1.417; *p* = 0.246; pη^2^ 0.058	F(1, 33) = 3.611; *p* = 0.066; pη^2^ 0.099
Interaction Task × Group		F(1, 24) = 5.967; ***p* = 0.022**; pη^2^ 0.245	F(1, 33) = 10.6; ***p* = 0.003**; pη^2^ 0.243	F(1, 25) = 5.809; ***p* = 0.024**; pη^2^ 0.189	F(1, 51) = 8.178; ***p* = 0.006**; pη^2^ 0.138	F(1, 23) = 6.017; ***p* = 0.022**; pη^2^ 0.207	F(1, 33) = 7.821; ***p* = 0.009**; pη^2^ 0.192

*For response times, means ± SEM are presented. pη^2^, partial η^2^. Bold p-values indicate significant effects (p < 0.05).*

**TABLE 3:** Multitasking costs (dual-task RT2—single-task RT) for the control and experimental groups in Experiment Series 1.

		Group					
		Dyslexia	Neuroticism	Nicotine deprivation	Video-gaming	Bi-lingualism	Coffee
Costs Controls	ms	685.509 ± 46.323	636.068 ± 31.774	879.829 ± 57.987	820.818 ± 52.645	912.652 ± 77.018	677.194 ± 43.070
Costs Group	ms	934.138 ± 90.747	870.495 ± 55.661	1,086.161 ± 42.645	646.352 ± 33.849	683.925 ± 48.263	536.724 ± 26.226
Difference	ms	248.629	234.427	206.333	−174.466	−228.727	−140.637
Difference	%	36.269	36.856	23.452	−21.255	−25.062	−20.768

*For costs, means and SEM are given. Difference is calculated with Controls as reference. For instance, dyslexics show 248 ms, i.e., 36%, higher multitasking costs than non-dyslexics.*

It is conceivable that some EF demands occur before or at the stage of the processing bottleneck (e.g., inhibition of task 2), while other EF demands occur after the bottleneck processing of the first task has been finished (e.g., switching the bottleneck to task 2, De Jong, 1995; for a more detailed argument including a graphical illustration see Supplementary Information). To estimate the costs before or at the bottleneck, we calculated (dual-task RT1 – single-task RT) for each participant individually and then compared the groups, i.e., (dual-task RT1 – single-task RT)_Experimental group_ – (dual-task RT1 – single-task RT)_Control group_, which is equivalent to the interaction term in the above analyses, except that now RT1 is used instead of RT2. To estimate the costs in task 2 after the bottleneck we calculated (dual-task RT2 – dual-task RT1)_Experimental group_ – (dual-task RT2 – dual-task RT1)_Control group_. Table 4 shows the group differences in multitasking costs for each type of cost separately. It shows that about two thirds of the overall group differences in multitasking costs occurred before or at the stage of the bottleneck, while about one third occurred in task 2 after bottleneck processing in task 1 has finished. In other words, results show that inter-individual differences in EF capabilities affect task processing before, at and after bottleneck processing, suggesting that EF are demanded at several stages during the processing of a dual-task, with the majority of demands occurring before or at the stage of the bottleneck.

**TABLE 4:** Detailed analysis of multitasking costs in Experiment Series 1.

	Group						
		Dyslexia	Neuroticism	Nicotine deprivation	Video-gaming	Bi-lingualism	Coffee
Group differences in overall costs	ms	248.629* (100%)	234.427** (100%)	206.333* (100%)	−174.466** (100%)	−228.727* (100%)	−140.637* (100%)
Costs before and at the bottleneck	ms	149.203* (60.010%)	163.374* (69.691%)	157.107* (76.142%)	−119.168* (68.305%)	−134.225 (58.684%)	−97.063* (69.017%)
Costs in task 2 after the bottleneck	ms	99.427* (39.990%)	71.054 (30.310%)	49.226* (23.858%)	−55.297* (31.695%)	−94.502* (41.317%)	−43.574 (30.098%)

The group differences in overall costs as used in the main analyses are split into the costs occurring before or at the stage of the bottleneck and those occurring in task 2 after the bottleneck. One-sample t-tests vs. 0 (i.e., no costs) *p < 0.05; **p < 0.01. All costs refer to the group differences in multitasking costs.

To assess whether the groups also differed in their single-task performance alone, we calculated independent-samples *t*-tests comparing the single-task RTs of the experimental groups with those of the control groups, individually for each experiment. Results showed that no statistically significant difference was evident for the studies on dyslexia [for all comparisons Group > Controls; 78.141 ms; t(24) = 1.684; *p* = 0.105; Cohen’s *d* = 0.687], neuroticism [32.691 ms; t(33) = 1.310; *p* = 0.196; Cohen’s *d* = 0.459], bilingualism [29.054 ms; t(23) = 0.664; *p* = 0.513; Cohen’s *d* = 0.277], and coffee consumption [−12.232 ms; t(33) = 0.520; *p* = 0.606; Cohen’s *d* = 0.181], while the difference was significant in the study on video-gaming [−65.502 ms; t(51) = 2.651; *p* = 0.011; Cohen’s *d* = 0.742] and in the study on smoking deprivation [183 ms; t(25) = 3.046; *p* = 0.005; Cohen’s *d* = 1.218]. Overall, this shows that the experimental and control groups performed comparably in the single-task conditions in four of the six studies. Note that differences in single-task performance cannot account for the further above reported interaction effects.

For the analyses of error rates, trials were classified as either a correct trial, or as an error trial, irrespective of whether there were multiple errors within a single trial (which was possible in dual-task trials). 2 × 2-factorial ANOVAs of the error rates, comparable to those of the response time analyses above, revealed either non-significant differences, or differences of the same nature, i.e., slower response times were accompanied by higher error rates. The only exception was the study on video-gaming, in which the video-gamers showed significantly higher dual-task costs in error rates than the non-gamers. Full details of error-rate analyses can be found in the Supplementary Information.

### Discussion Experiment Series 1

Results showed that the multitasking costs varied across groups as predicted by their presumed executive function capabilities, supporting the active scheduling account (Marois and Ivanoff, 2005; Sigman and Dehaene, 2005).

For some of the groups we compared, there is an ongoing discussion in the literature whether it is indeed the proposed underlying factor which causes differences in the EF capabilities, or whether it can be explained otherwise. For example, it might be that frequent video-gamers differ in a number of characteristics from non-gamers, and that it is not only video-gaming experience, but also some of these further characteristics which may explain part of the differences in EF capabilities. While Bediou et al. (2018) in their meta-analysis indeed provided evidence for this, they at the same time showed that such additional characteristics cannot fully explain the difference between gamers and non-gamers. However, importantly, this is not a major issue for the present study, because here it is only relevant that the groups differ in their EF capabilities, but not why exactly they differ. Thus, the present study can (to some extent) remain oblivious to the in-depth discussions about the true nature of the underlying mechanisms of EF differences.

However, group comparisons bear the natural limitation that there might be further differences besides the postulated differences in executive function capabilities. While this is likely to be the case when looking at an individual group, we believe that the only common difference across all six groups is a difference in executive functions (see section “General Discussion” for a more in-depth discussion). Nevertheless, to rule out the possibility that the current findings are explained by a potential further factor common to all groups, Experiment Series 2 was conducted, which was not based on between-group comparisons, but instead on correlational within-subject designs.

## Experiment Series 2

Experiment Series 2 aimed at assessing the EF capability of each participant individually and correlating it with their multitasking ability. To assess EF capabilities, we used complex working memory span tasks (Redick et al., 2012). Complex working memory span tasks were designed to assess the so-called working memory capacity (WMC). Theoretical models (Kane et al., 2007) as well as empirical data (McCabe et al., 2010) suggest that WMC is closely related, if not identical, to the concept of executive functions. In more detail, Kane et al. (2007) considered the central executive, working memory capacity, executive attention, and supervisory attention system as synonymous. In support of this assumption, McCabe et al. (2010) measured working memory capacity (four different complex working memory span tasks) and executive functions (Wisconsin Card Sorting Test, verbal fluency, mental arithmetic, mental control), and found that the two resulting factors in a factor analysis correlated extremely high with each other (*r* = 0.97). The authors conclude that both, working memory capacity and executive functions, share an underlying attentional ability, which they term executive attention (Engle and Kane, 2004; McCabe et al., 2010). Therefore, we assume that complex working memory span tasks assess executive functions.

Working memory capacity is assessed by complex working memory span tasks, such as the reading span task, in which a processing task (e.g., determine the correctness of a sentence) is intermingled with a short-term memory task (e.g., memorizing letters). To create a reliable picture which is independent of a specific domain, we used three different versions of this task: (a) the classic reading span task, relying on linguistic processing, (b) the symmetry span task, relying on visual pattern-recognition processing, and (c) the rotation span task, relying on spatial processing (Daneman and Carpenter, 1980; Foster et al., 2015). Multitasking ability was again assessed in form of multitasking costs, i.e., performance in the dual-task minus performance in the single-tasks.

If the passive queuing account holds, which proposes that the presence of a bottleneck does not demand additional executive functions, then working memory capacity should be independent of multitasking costs, i.e., no correlation is expected. However, if the active scheduling account holds, then higher working memory capacity should result in more efficient processing in the multitask at the stage of the bottleneck and, consequently, the behavioral multitasking costs should be lower, i.e., a negative correlation between span scores and multitasking costs is expected.

### Methods

#### Overview of Tasks

All three reported experiments were final year undergraduate dissertation projects conducted at Brunel University London between 2013 and 2017, supervised by the first author, AJS. All students worked independently and tested independent samples. The studies were run by the following students: Reading Span: KT; Symmetry Span: KK; Rotation Span: BS.

#### Participants

All studies were individually approved by the Department of Life Sciences Ethics committee, Brunel University London, and were carried out in accordance with the Declaration of Helsinki. Participants gave written informed consent before participation and were debriefed after the study. Participants were either reimbursed with course credits, £10, or volunteered for free. Virtually all participants were students of Brunel University London between the age of 18 and 25.

In total 85 participants took part (Table 5). We excluded participants as in Experimental Series 1, which resulted in the exclusion of 4 participants. More details on participant selection can be found in the Supplementary Information.

**TABLE 5:** Demographic information for Experiment Series 2.

	Experiment		
	Rotation span	Symmetry span	Reading span
N Original	29	20	36
N Final	27	20	34
N females	13	13	25
N males	14	7	11
Age mean	22.370	20.625	19.556
Age range	20–32	18–27	18–25
Age s.d.	2.924	1.784	1.520

*N Original refers to participant numbers before selection, N Final to number after selection. Age data refers to the final sample and is given in years. s.d., standard deviation.*

#### Design

All studies in Experiment Series 2 were based on a correlational design in which each participant performed the dual-task as well as one type of complex working memory span task. The outcome variables of the multi-task were the multitasking costs calculated as (a) dual-task RT2 minus single-task RT, and (b) error-rate dual-task minus error-rate single-task. The outcome variables in the different span tasks were the respective partial scores, i.e., the number of items correctly recalled in serial position.

#### Materials and Procedure

The PRP dual-task was identical to Experiment Series 1, except that, to shorten the experiment, the dual-task was performed only in one task order (first auditory task, then visual task). The visual stimuli were always the numbers 1 and 2, and the auditory stimuli always the low- and high-pitched tones (see Methods Experiment Series 1). A session, including the dual-task and working memory span task, lasted 60–70 min.

In all complex working memory span tasks, a so-called processing task was interwoven with a memory task (Figure 1, right panel). The nature of the tasks varied across the experiments, and followed the procedure as described in Foster et al. (2015). In the symmetry span task, participants had to decide whether a pattern of black boxes in a matrix is symmetrical around its vertical axis or not (processing task) and to memorize the position of a red square in a 4 × 4 matrix (memory task). In the rotation span task, participants had to decide whether a rotated letter is in its normal form or whether it is mirror reversed (processing task) and to memorize an arrow which could be either short or long and point to one of eight directions (memory task). In the reading span task, participants had to read sentences and decide whether they made sense or not (processing task) and to memorize letters (memory task). One memory trial always started with the presentation of one processing task (e.g., one sentence in the reading span task). After the processing task has been shown (e.g., a sentence), another screen appeared (not shown in Figure 1) on which participants gave their response to the processing task using the computer mouse. For this, a question was asked (e.g., “Does this sentence make sense?”) with two clickable answer boxes (Yes and No). After the response to the processing task (or a time-out error message), one item to be remembered was presented (e.g., one letter in the reading span task). This cycle was repeated randomly anywhere from two up to seven times (depending on the task). At the end of one memory trial, participants had to recall all items to remember (e.g., letters) in the order of their presentation. For this, they used the computer mouse and clicked either on the appropriate squares in a 4 × 4 matrix (symmetry span task), or on the appropriate arrows in a display of all 16 arrows (short and long in 8 directions; rotation span task), or on the appropriate letter in a matrix of all potential letters (reading span task). To avoid that participants traded off performance in the processing task to rehearse the memory items, they were asked to maintain accuracy in the processing task above 80% and had an individually adjusted time window in which the response had to be given before a time-out error was presented.

### Results

The aim of these experiments was to show that individual differences in executive function capabilities are associated with multitasking abilities on a subject-by-subject level. For this, we correlated dual-task costs (response time of the second task in the dual-task (RT2) minus single-task RT) with different measures of working memory capacity which reflect executive function capabilities. While the passive queuing account predicts no correlations, the active scheduling account predicts a negative correlation, i.e., the higher the working memory span the lower the dual-task costs. Due to this strongly directional hypothesis (to the best of our knowledge, there exists no model predicting a positive correlation), we applied one-sided significance testing.

**FIGURE 3:**
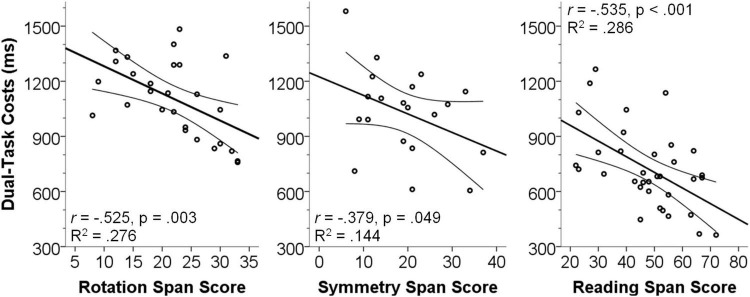
Scatterplots illustrating the association between dual-task costs and measures of working memory capacity. Dual-task costs (ms) were calculated as dual-task response times (RT2) minus single-task response times. Span scores are the raw partial scores. Error lines show the SEM of the best line of fit.

Results (Figure 3) showed that in all three studies dual-task costs in terms of response times were significantly negatively correlated with the respective span scores (Rotation span: *N* = 27, Pearson’s *r* = −0.525, *p* = 0.003, R^2^ = 0.276; Symmetry span: *N* = 20, Pearson’s *r* = −0.379, *p* = 0.049, R^2^ < 0.144; Reading span: *N* = 34, Pearson’s *r* = −0.535, *p* < 0.001, R^2^ = 0.286). Although we already correlated the dual-task costs, which are a relative measure not directly dependent on generic processing speed of an individual, with the partial span score, we confirmed above analyses by partialing out the single-task performance as a measure of processing speed. Results showed the same pattern (all *p* < 0.05; all Pearson’s *r* < −0.393), illustrating that the observed correlations are truly linked to dual-task costs and not caused by correlations between working memory capacity and generic processing speed.

Comparable correlations of dual-task costs in terms of error rates and the span scores (see Supplementary Information for details) also showed negative correlations which, however, mostly were not significant (Rotation span: *N* = 27, Pearson’s *r* = −0.249, *p* = 0.105, R^2^ = 0.062; Symmetry span: *N* = 20, Pearson’s *r* = −0.026, *p* = 0.456, R^2^ < 0.001; Reading span: *N* = 34, Pearson’s *r* = −0.399, *p* = 0.009, R^2^ = 0.159).

To describe the relationship between working memory capacity and response-time dual-task costs in more practical terms, we split the span scores into quartiles (individually for each span task) and calculated the mean span score and the mean multitasking costs for the lowest and highest quartile. These values therefore reflect the average performance of low-span (lower quartile) and high-span (upper quartile) participants of each sample, respectively. Results (Table 6) showed that low-span participants had between 148 ms and 307 ms higher dual-task costs as compared to high-span participants, which is equivalent to an increase of between 16% and 51%.

**TABLE 6:** Illustration of the effect sizes in Experiment Series 2.

Span task	Span score low	Span score high	Difference span scores	Costs low (ms)	Costs high (ms)	Difference costs (ms)
Rotation Span	11.5	31.5	20 (−63.49%)	1,215.123	931.185	283.928 (30.49%)
Symmetry Span	9	31.8	22.8 (−71.70%)	1,078.668	930.862	147.806 (15.88%)
Reading Span	28	65	37 (−56.92%)	909.595	602.389	307.206 (51.00%)

*For each study, participants were divided into quartiles based on their respective span scores. Data of the lowest (low) and highest (high) quartiles are shown. Span scores reflect raw partial scores, and costs reflect multitasking costs (dual-task RT2—single-task RT). For calculation of percentages the high span group served as reference; for instance, in the rotation span the low group had 63% lower scores in the span task than the high group and showed 30% higher dual-task costs.*

In Experiment Series 1 we found that multitasking costs arising before, at and after the bottleneck were affected by inter-individual differences in EF capabilities. To corroborate this finding, we first correlated the span scores with dual-task RT1 costs (dual-task RT1 – single-task RT) which reflect the costs before and at the bottleneck. Because we tested for a very specific and fine-grained effect for which we had a directed hypothesis, we used one-sided significance testing. Pearson’s correlations were significantly negative in the rotation span (*r* = −0.505, *p* = 0.004, R^2^ = 0.255) as well as the reading span (*r* = −0.409, *p* = 0.008, R^2^ = 0.167), while the correlation only approached significance in the symmetry span (*r* = −0.322, *p* = 0.084, R^2^ = 0.104). To test for costs arising in task 2 after the bottleneck, we correlated the span scores with a measure of dual-task RT2 – dual-task RT1 (see Supplementary Information for details). Correlations were significantly negative in the symmetry span (*r* = −0.386, *p* = 0.046, R^2^ = 0.149) and the reading span (*r* = −0.355, *p* = 0.020, R^2^ = 0.126), while the rotation span was not significant (*r* = −0.013, *p* = 0.434, R^2^ = 0.0002). We take the finding that five out of the six correlations were significant or approaching significance as support of our conclusion from Experiment Series 1, i.e., that inter-individual differences in EF capabilities affect task processing before, at, and after the stage of the bottleneck, suggesting that EF are demanded at several stages during the processing of a dual-task.

### Discussion Experiment Series 2

Results again support the active scheduling hypothesis and demonstrate that individual EF capabilities are predictive of multitasking performance. Working memory span tasks are considered to be very good measures of EF capabilities (Kane et al., 2007; McCabe et al., 2010), so that the findings of Experiment Series 1 and 2 combined are genuinely related to EF, and not to other potential co-varying functions.

## General Discussion

In Experiment Series 1 we have shown that all three groups of participants who are known to have impaired executive function capabilities (dyslexics, high neurotics, nicotine deprived) showed higher multitasking response-time costs as compared to the respective control groups. Along the same lines, all three groups of participants who are known to have improved executive function capabilities (video-gamers, bilinguals, coffee consumers) showed lower costs as compared to the respective control groups. In Experiment Series 2 we have shown that working memory capacity as assessed by the rotation span, the symmetry span, and the reading span tasks, is negatively correlated with multitasking costs, i.e., higher working memory capacity is associated with better multitasking performance. These results, derived from nine independent studies, provide strong evidence for an active scheduling account of bottleneck processing in multitasking and demonstrate that inter-individual differences in executive function capabilities affect multitasking performance.

One component of the multitasking costs is caused by the pure waiting of the second task until the bottleneck is freed up from first task processing (the refractory period) (Pashler, 1994). We have shown the existence of a second component which is caused by the EF required to coordinate task processing at the stage of a bottleneck (Marois and Ivanoff, 2005) and that the amount of these second costs is closely related to the individuals’ EF capabilities.

The observed effects are quite profound in size. For instance, the multitasking costs of dyslexics were 248 ms higher than those of non-dyslexics, and the difference in multitasking costs between low and high span participants in the reading span task was 307 ms. Again, transferred to a car traveling at 30 mph, this would increase the braking distance by 3.3 m and 4.1 m, respectively. Therefore, we believe that it is of high importance to design human-machine interfaces, including car cockpits and graphical user interfaces, in a way which avoids concurrent demands on mental mechanisms subject to the central attentional bottleneck.

The above numbers allow for a tentative estimation of the relative contributions of each component, i.e., refractory waiting time and executive functions. In Experimental Series 1, the experimental groups showed higher or lower multitasking costs by between 140 ms and 248 ms, i.e., between 20% and 36% of the total multitasking costs. Taking into account that this is only a modulation of demands on executive functions, and that executive functions also explain part of the multitasking costs in the control groups, it seems legitimate to assume that for the experimental groups, the above numbers constitute a lower limit of the contribution the demands on executive functions make to multitasking costs. This shows that a profound proportion of the delays caused by a central attentional bottleneck is actually caused by executive functions coordinating task processing, and not by a mere refractory waiting of the second task.

Further scrutiny revealed that the group differences in multitasking costs did not arise from a single point in the processing chain, but instead affected task processing at several stages. With the current data, we were able to show that processing is affected before and/or at the bottleneck stage, as well as after the bottleneck stage. This is in agreement with suggestions that a number of EFs are needed at various stages to process dual-tasks suffering from a bottleneck, such as preparation, order-scheduling, inhibition, switching, and monitoring (see Supplementary Information). We conclude that EF seem to exert a very tight control over most, if not all, processing in multitasking.

The current study does not allow insight into the exact nature of the executive functions involved in coordinating task processing at the stage of the bottleneck. Thus, in principle our theoretical assumptions and results are in agreement with most models proposing additional coordinative demands, whether expressed as more traditional EFs such switching and inhibition, as setting of task parameters (Meyer and Kieras, 1997; Logan and Gordon, 2001), as control of hierarchically higher task structures (Hirsch et al., 2017, 2018), as re-distribution of resources in capacity sharing models (Navon and Miller, 2002; Fischer and Plessow, 2015), or as resolution of interference due to cross-talk (Koch, 2009). However, as argued in the previous paragraph, the current findings suggest that it is not only a single stage in task processing where EFs are involved, but several stages.

There have been suggestions that dual-tasking constitutes an EF on its own, separate from EFs such as switching, updating, and inhibition (Miyake et al., 2000; Strobach et al., 2014), although strong evidence in support of this is sparse. Opposed to these suggestions, we recently have shown that the additional processes demanded in PRP tasks are strongly linked to the EF of working memory (Otermans et al., 2021), and, in an fMRI study, that there is profound overlap in the brain areas associated with dual-task coordination (PRP task), inhibition (Stroop task), switching (Task switching), and updating (n-back task) (Saylik et al., in press). But even if dual-tasking is considered a distinct EF, this does not mean that a specific implementation of a dual-task, such as the PRP task, may not also demand other EF, such as switching, inhibition, and/or updating. Therefore, the existence of a distinct dual-tasking EF would not be in disagreement with the current findings and our interpretations.

While we may not be able to specify the involved EFs in detail, including the question of whether dual-tasking is a separate EF, we would like to note that this is not the main aim of the present study. In more detail, we aimed at providing further evidence whether a central attentional bottleneck creates additional mental demands (active scheduling account) or not (passive queuing account). Our findings, derived from nine different studies, support the active scheduling account. Based on prior literature and the present study, we believe that EF in the traditional sense, i.e., inhibition, switching, updating, and potentially dual-tasking, are the most likely candidate processes for these additional mental demands. However, other closely related concepts, such as controlled or executive attention (Kane et al., 2007) or other hierarchical action-coordinating mechanisms (De Jong, 1995; Hirsch et al., 2018) would result in the same prediction. In our view, the exact nature of the additional mental demands is not of key relevance in this study, because the active scheduling account would still be supported in the same way (but would suggest that further studies are needed to refine the nature of the underlying processes, e.g., Otermans et al., 2021). While further factors beyond EFs and/or controlled attention might be conceivable, it is noteworthy that only factors affecting specifically the multitasking situation can explain the current findings (meaning that such a factor either affects only the multitasking situation, or affects the multitasking situation over-additively stronger than the single-tasks). This is due to the interaction analysis (and in particular the even stricter test detailed in section 2.1 of the Supplementary Information). Therefore, generic more low-level factors such as processing speed cannot account for the current findings, and we suggest that only hierarchically more higher-level factors can explain our findings.

In all experiments, the response times to the first task in the dual-task (RT1) were higher than the single-task response times, which is in slight disagreement with a strict bottleneck model. This finding may have been caused by response grouping, i.e., withholding R1 until R2 is ready (Pashler, 1994). In addition, the prolonged RT1 as compared to single-task performance (which are the RT1 costs) were modulated by the experimental manipulations and correlated with the WM capacity. We believe that this is caused by demands on EF at or before the bottleneck stage, such as the coordination of higher-level task representations (Hirsch et al., 2017, 2018).

The fact that concurrent demands on the central attentional bottleneck not only results in deferment of at least one task, which in itself is already problematic, but also demand EF has wide-reaching implications for everyday life, in which multitasking has become an ubiquitous phenomenon in private and work contexts. First, it implies that the EF are partially or even fully occupied by bottleneck task scheduling so that during this time (estimated to be at least 150–250 ms) they are not available for other tasks, which might amplify potential problems in critical situations, such as introducing a braking and obstacle avoidance maneuver at the same time while driving. Second, in particular in work contexts, frequent concurrent demands on the attentional bottleneck imply frequent demands on EF functions, which may promote the development of mental fatigue and stress, both of which have been shown to negatively impact EF, possibly resulting in a viscous cycle (van der Linden et al., 2003). Third, the current findings may provide one explanation for the well-known link between multitasking abilities and proneness to falling in the elderly (Herman et al., 2010). Finally, our results show that these problems may be amplified for certain parts of the population, such as dyslexics, highly neurotics, smokers, and possibly many more groups, making those groups even more prone to being mentally slowed down in critical situations and to develop mental fatigue and experience stress.

## Data Availability Statement

The data are now available via Figshare: Doi: 10.17633/rd.brunel.14554902.

## Ethics Statement

The studies involving human participants were reviewed and approved by Department of Life Sciences Ethics Committee, Brunel University London. The patients/participants provided their written informed consent to participate in this study.

## Members of the Brunel Students

Caitlin Ball, Jessica Boyce, Mark Buckley, Rahmi Saylik, Nargis Ghani, Ayan Omar, Luwam Simon, Asli Senkoy, Kirti Kumar, Barry Smith, and Kai Tyler.

## Author Contributions

AJS designed all studies, conducted all data analyses, and wrote the manuscript. CB, JB, MB, RS, NG, AO, LS, AS, KT, KK, and BS contributed to the design, performed the data collection, and contributed to the data analyses.

## Conflict of Interest

The authors declare that the research was conducted in the absence of any commercial or financial relationships that could be construed as a potential conflict of interest.

## Publisher’s Note

All claims expressed in this article are solely those of the authors and do not necessarily represent those of their affiliated organizations, or those of the publisher, the editors and the reviewers. Any product that may be evaluated in this article, or claim that may be made by its manufacturer, is not guaranteed or endorsed by the publisher.

## Footnotes

1 Please note that for our arguments it is only important that serial processing occurs, but that it is irrelevant whether this serial processing is caused by a strategic or a structural bottleneck (Fischer and Plessow, 2015), or at which processing stage(s) it occurs. We do not assume that a structural bottleneck necessarily means processing based on the passive first-come first-served approach, but that also a structural bottleneck may require additional control, e.g., to coordinate the processing order of the tasks.

2
www.neurobs.com
