# Supplementary material for: Inter-Individual Differences in Executive Functions Predict Multitasking Performance – Implications for the Central Attentional Bottleneck
Source: Front Psychol. 2022 May 11;13:778966. doi: 10.3389/fpsyg.2022.778966 (PMC9131123; doi:10.3389/fpsyg.2022.778966)
Supplement: Supplementary file 1 [file Data_Sheet_1.pdf]

Supplementary Information for

Inter-individual differences in executive functions predict multitasking performance – Implications for the central attentional bottleneck

André J. Szameitat

Corresponding Author: *Andre Szameitat*
Email: *Andre.Szameitat@Brunel.ac.uk*

This PDF file includes:

Supplementary text
Figures S1 to S3
Table S1
SI References

1. Methods – Experiment Series 1

1.1. Group characterizations

In the following, a detailed description of how the experimental groups were defined is presented. In addition, detailed information about excluded participants is given.

Dyslexia. Students suffering from dyslexia were recruited using Brunel University's Dyslexia and Disability Service (DDS). Participants were classified as dyslexic when they confirmed that they had been diagnosed as being dyslexic by some form of authority, school, health service, or the DDS. We did not differentiate between different types of dyslexia. Non-dyslexic participants were recruited among the remaining student population and they had to confirm that they had never been diagnosed with dyslexia and that they do not suffer from symptoms which would indicate the presence of dyslexia. Three participants in the dyslexic group and five participants in the non-dyslexic group showed error rates above 30% and were excluded.

Neuroticism. To create extreme groups of high- and low-neurotics, we screened 400 participants using the 24-item neuroticism scale of the Eysenck Personality Questionnaire (EPQ; Eysenck & Eysenck, 1975). From those screened, 39 were selected based on their EPQ scores: 22 (11 female) were in the High-N group (mean EPQ score=18, range=16–24) and 17 (9 female) were in the Low-N group (mean EPQ score= 3.89, range=0–6). All of the participants were right-handed as assessed by the Edinburgh Inventory (Oldfield, 1971) and had normal or corrected to normal vision. Each participant gave written informed consent and was paid £10. Three participants in the low-neuroticism group and two participants in the high-neuroticism group showed error rates above 30% and were excluded.

Nicotine deprivation. Only regular smokers (N = 60) took part in this study. Participants in the nicotine-deprivation group (N = 31) were asked to refrain from smoking for at least 30 min before the experiment. This time was chosen to facilitate participant recruitment and ensure compliance with the instructions. Considering the time it took to fill out forms, read instructions, and practice the task, there were at least 50-60 min between the last smoking and the start of the proper experiment. Participants in the non-deprived group (N = 29) were asked to smoke their typical type of cigarette directly before the session started. To confirm level of perceived nicotine deprivation, participants filled out a brief questionnaire directly after the study had finished. In more detail, they had to indicate their urge to smoke on a scale from 1 (no urge at all) to 10 (very strong urge). We considered participants who provided a score of 8 or higher for the deprived group and those who provided a score of 3 or lower for the non-deprived group. This resulted in the exclusion of 18 participants (14 in the deprived group and 4 in the non-deprived group) which indicated that many participants did not feel high levels of urge to smoke after such a comparatively brief duration of nicotine abstinence. Two further participants were excluded due to missing data in the questionnaire, leaving 40 participants.

Of those 40 participants, seven participants of the nicotine deprived group and six participants of the non-deprived group were excluded due to error rates above 30%, leaving the final number of 27 participants (10 deprived, 17 non-deprived) for analyses.

After the above selection procedures, the nicotine deprived group (N = 10) reported an average deprivation score of 8.550 (range 8 – 9.5, s.d. 0.497), and the non-deprived group (N = 17) reported a score of 1.971 (range 1 – 3, s.d. 0.649).

Video-gaming. Participants were recruited, among other routes, via the Union of Brunel Students' gaming society. All participants filled out a questionnaire asking how many hours per day they spend on average gaming any type of games, and in addition how much time of that they spent playing action games. Action games were defined as games emphasizing physical abilities, reaction time, hand-eye coordination and reflexes, such as first-person shooter games, racing simulations, etc. The gamers spend on average 5.196 hours (s.d. 2.575 hours) per day playing any kind of game, and of that they spend 3.5 hours (s.d. 1.991) on action games. The non-gamers spend 1.346 (s.d. 2.732) hours per day, of which were 0.615 (s.d. 1.446) hours on action games. A non-parametric Mann-Whitney U test for two independent samples confirmed that gamers spend significantly more time playing games than non-gamers in general ($U = 30$; $Z = 3.958$; $p < .001$) and in action games ($U = 27$; $Z = 4.096$; $p < .001$). Seven participants in the video-gamer group and five participants in the non-gamer group showed error rates above 30% and were excluded.

Bilingualism. We defined bilingualism as being able to speak at least two languages with the proficiency of a native tongue by the age of six years. Monolingualism was defined as having only one native tongue. Languages learned later in life, for instance as part of education in school, were not considered. We used a questionnaire and self-report to assign participants to either group. Three participants in the bilingual group and four participants in the monolingual group showed error rates above 30% and were excluded.

Coffee. All participants were asked to not consume any caffeine for at least four hours before the study commenced. After having read the information sheet and having given informed consent, participants in the coffee group drank a cup of coffee (200 ml) made out of 3 teaspoons of standard soluble coffee granules and hot water (no further ingredients such as

sugar, sweeteners, or milk were used). The non-coffee group did not receive any drink. Participants then had to fill out further forms and practice the task. The main experiment started approx. 30 min after the coffee consumption. One participant of the coffee group had dual-task costs which deviated by 3.651 standard deviations (i.e., more than the 2.5 as used as a criterion) from the group mean and was excluded. Four participants in the coffee-consumption group and five participants in the non-coffee group showed error rates above 30% and were excluded.

2. Results - Experiment Series 1

2.1. Additional analyses on the interaction analyses

One might argue that the interactions depicted in Fig. 2 of the main article are a result of a mere prolongation of the response selection stage in the affected groups. For instance, suppose that dyslexics are slower in selecting a response as compared to non-dyslexics. This might explain why dyslexics are slower in the single-tasks by 78 ms as compared to the non-dyslexics (cf. Table 2 in the main article). In the PRP dual-task, the response selection stage has to be performed twice in serial order (once for each task), so that one would expect the dyslexics to be slower in the dual-task by 156 ms (78 ms per response selection stage) as compared to the non-dyslexics. This effect may already cause a significant interaction which, however, would be purely driven by the adding-up of a mere response selection slowing, but not by impaired executive functions. While it can be seen in Table 2 that already the numerical effects are much larger than predicted by a pure response-selection effect (e.g. in dyslexia the group difference in RT2 is 327 ms instead of 156 ms), we tested this statistically. For this, we took a highly conservative approach which reduced the difference between groups in the dual-task by the difference in the single-tasks. As an example, in the case of the dyslexia study, we did not test using a difference of 327 ms, but a difference of 249 ms (327

ms – 78 ms). In terms of a graphical representation, this is equivalent by bringing down the dual-task data point of the dyslexics (top right data point) by the amount of the difference between dyslexics and non-dyslexics in the single-tasks (i.e., 78 ms; the left two data points). Because this massively under-estimated the interaction term and we had clear directed hypotheses, we used one-sided significance testing. It turned out that even using such a conservative testing, four of the six interactions still were statistically significant according to our hypotheses (all p-values between .040 and .004), with the interaction for the dyslexia study approaching significance (p = .053). The interaction in the smoking study did not reach significance (p = .394). This is strong evidence that the observed interactions are not caused by an effect already present in the single-tasks which just adds up in the dual-task. Instead, we interpret the findings as a genuine effect of the respective group difference on the performance of a PRP dual-task.

2.2. Error rates

In the following, detailed analyses of the error rates are presented (Table S1 and Fig. S1).

Table S1. Descriptive and inferential statistics of error rates in Experiment Series 1.

		Group					
		Dyslexia	Neuroticism	Nicotine deprivation	Video-gaming	Bi-lingualism	Coffee
Measure	Unit						
Sample Size (Group)	N	14	20	9	31	12	20
Sample Size (Controls)	N	12	15	16	22	11	16
Single-Task Errors (Group)	%	6.528 ± 1.075	5.417 ± 0.836	7.407 ± 1.210	6.278 ± 0.629	6.667 ± 1.430	5.857 ± 0.801
Single-Task Errors (Control)	%	5.179 ± 0.669	5.500 ± 0.853	6.354 ± 1.080	5.812 ± 0.853	6.591 ± 1.100	4.018 ± 0.505
Multitask Errors (Group)	%	17.361 ± 1.37	11.583 ± 1.742	18.519 ± 2.167	14.578 ± 0.915	16.806 ± 2.377	10.714 ± 1.070
Multitask Errors (Control)	%	10.000 ± 1.260	9.889 ± 1.319	14.167 ± 1.887	10.877 ± 1.164	13.030 ± 1.841	12.321 ± 1.578
Main effect Task		F(1, 24) = 57.829; p <	F(1, 33) = 26.986; p < .001; pη^2 0.45	F(1, 23) = 51.452; p <	F(1, 51) = 82.424; p <	F(1, 21) = 35.76; p <	F(1, 34) = 75.057; p <

		.001; $p\eta^2$ .707		.001; $p\eta^2$ 0.691	.001; $p\eta^2$ 0.618	.001; $p\eta^2$ 0.63	.001; $p\eta^2$ 0.688
Main effect Group		F(1, 24) = 11.628; p = .002; $p\eta^2$ 0.326	F(1, 33) = .251; p = .620; $p\eta^2$ 0.008	F(1, 23) = 1.551; p = .225; $p\eta^2$ 0.063	F(1, 51) = 3.993; p = .051; $p\eta^2$ 0.073	F(1, 21) = .744; p = .398; $p\eta^2$ 0.034	F(1, 34) = .008; p = .931; $p\eta^2$ < .001
Interaction Task x Group		F(1, 24) = 8.529; p = 0.007; $p\eta^2$ 0.262	F(1, 33) = .765; p = .388; $p\eta^2$ 0.023	F(1, 23) = 1.563; p = .224; $p\eta^2$ 0.064	F(1, 51) = 4.854; p = .032; $p\eta^2$ 0.087	F(1, 21) = 1.781; p = .196; $p\eta^2$ 0.078	F(1, 34) = 5.147; p = 0.030; $p\eta^2$ 0.131

Note: *For error rates means ± SEM are presented. $p\eta^2$ = partial η^2.*

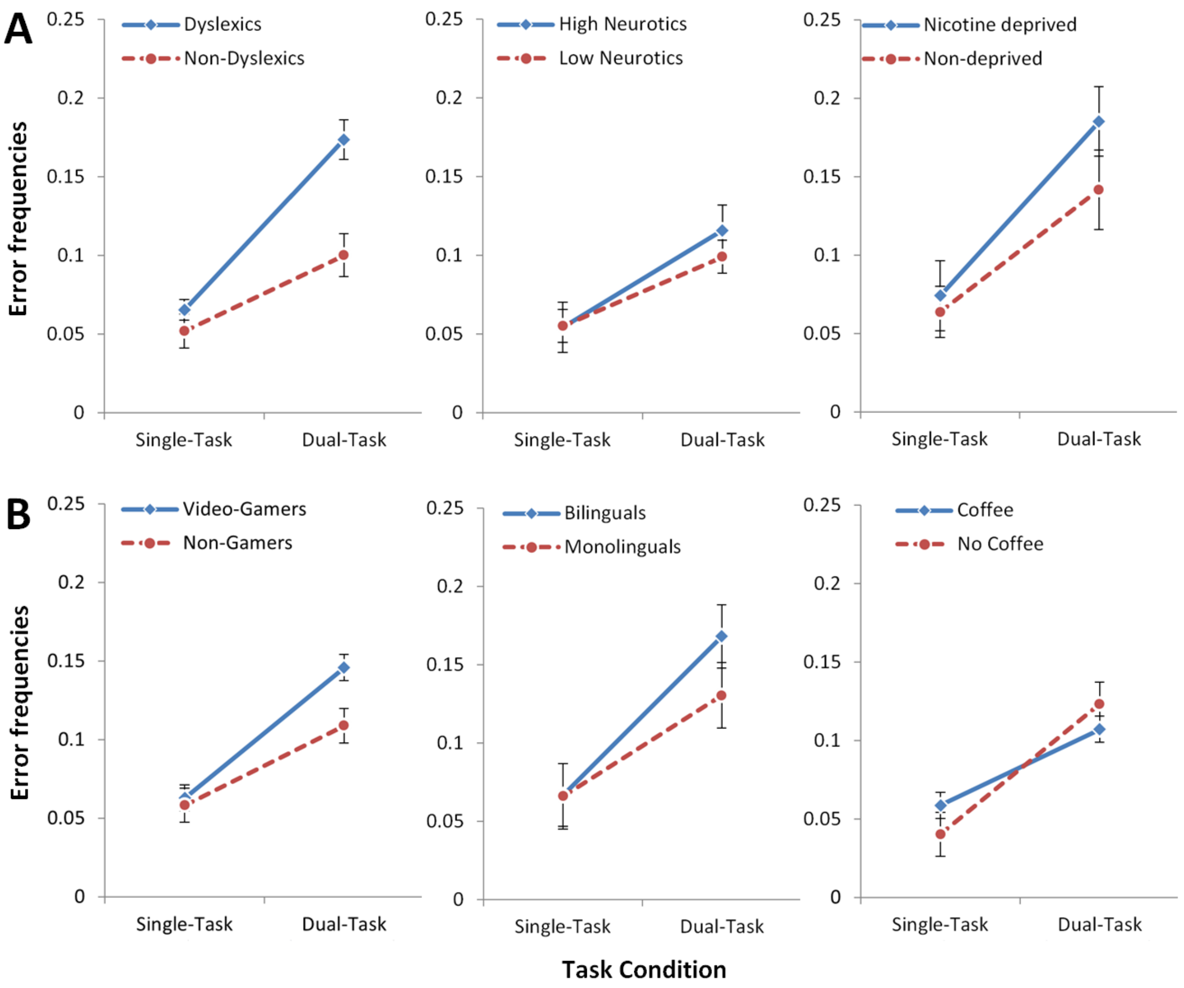

Fig. S1. Relative error frequencies for the three independent groups of participants who are known to have impaired executive functions (upper panel, A) and for the three groups

who are known to have improved executive functions (lower panel, B). Error bars show the standard error of the mean (SEM).

2.3.Executive functions in PRP dual-tasks (Experimental Series 1)

In the following, the executive functions are disentangled and described in a higher level of detail than in the main manuscript. Part of this discussion could also be presented in the main manuscript, pending editor/reviewer views and space allowance.

When considering a processing bottleneck, the need for executive functions may arise at different stages. For choice-response tasks, the processing chain is typically subdivided into three successive stages, the perception, response selection, and motor execution (Supplementary Fig S2). The response selection is a decisional process demanding controlled attention and is considered to constitute a bottleneck, i.e. it is limited to serial processing. Note, that for the current discussion we only need to assume that a bottleneck is present, irrespective of which stage(s) constitute the bottleneck and whether it is strategic or structural in nature.

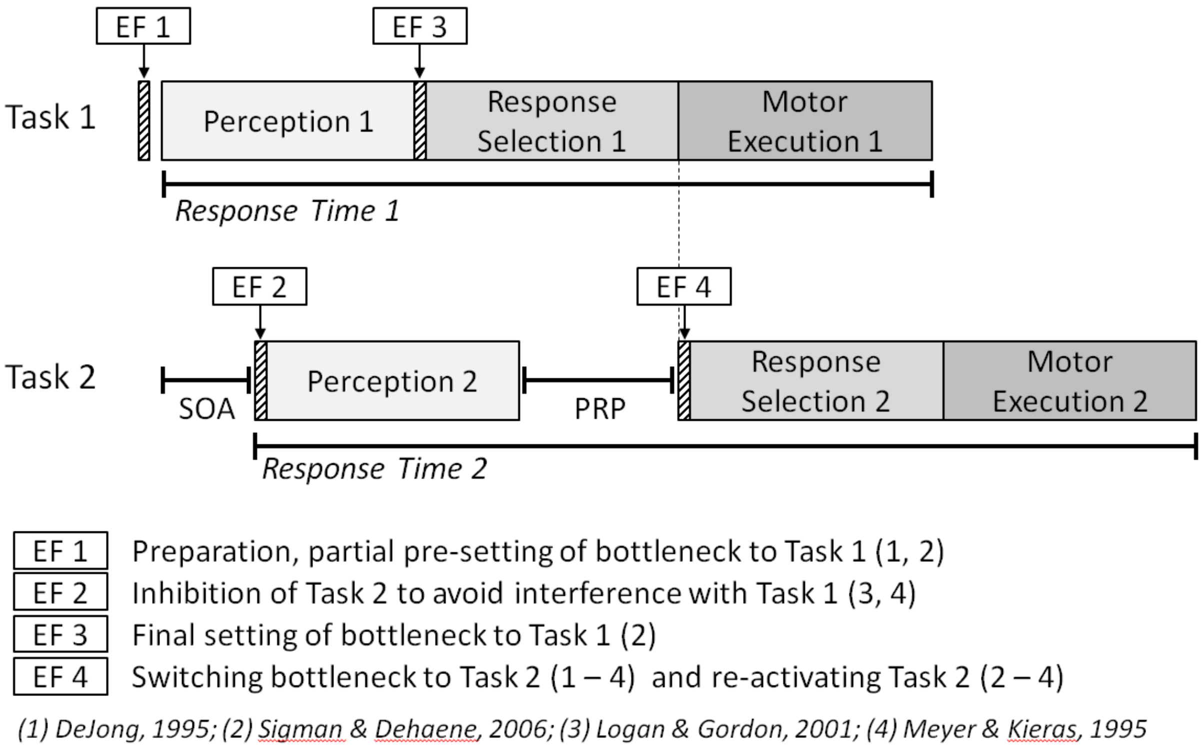

Fig. S2. Processing stages in a PRP dual-task. When two speeded choice-response tasks (Task 1 and Task 2) have to be performed simultaneously (Stimulus Onset Asynchrony, SOA, 0 ms) or rapidly after each other (SOA > 0 ms), research has shown that the processing stage of the response selection can work only serially, i.e. it constitutes a processing bottleneck. As a consequence, the response selection of Task 2 has to wait until the response selection of Task 1 has finished (refractory period). Peripheral stages such as perception and motor execution have been shown to mostly work in parallel.

Previous literature suggested the involvement of EF at various stages in task processing. First, it has been suggested that certain preparatory processes take place before the first stimulus is even presented (EF 1 in Fig S2), e.g. the preparation of the upcoming processing order (De Jong, 1995; Luria & Meiran, 2003). However, because they occur before stimulus 1 onset, they do not affect the response times and therefore can be ignored here (but they

might be assessed by brain imaging, e.g. (Szameitat, Lepsien, von Cramon, Sterr, & Schubert, 2006; Szameitat, Schubert, & Müller, 2011; Szameitat, Schubert, Müller, & von Cramon, 2002)). Second, there is evidence pointing to the fact that the preparation taking place in EF 1 is not complete, but instead that the task set can be implemented fully only once the stimulus has been presented (Monsell, 2003). As a consequence, when the perceptual processes of Task 1 have finished, the bottleneck is still not fully set to process Task 1 (Sigman & Dehaene, 2005). This final setting is presumable implemented by EF, as illustrated by EF 3 in Fig S2. Third, it has been suggested that the second task has to be actively inhibited (EF 2 in Fig S2) to avoid interference with task 1 processing (Logan & Gordon, 2001; Meyer & Kieras, 1997). Finally, when the bottleneck has finished processing task 1, it has to be switched to task 2 (EF 4) (De Jong, 1995; Sigman & Dehaene, 2005). In addition, it has been suggested that the initially inhibited task 2 needs to be activated or re-instantiated again to be processed (also EF4) (Logan & Gordon, 2001; Meyer & Kieras, 1997). Presently it is unclear how much each of these EF components (EF 2 – 4) might contribute towards multitasking costs, in particular because some suggestions have been made on the basis of more theoretical models and not empirical data (Logan & Gordon, 2001; Meyer & Kieras, 1997). By defining multitasking costs as RT2 – single task RT, the initial analyses of the main article (Results Experimental Series 1) assessed all components together.

However, it is possible to disentangle the components to some degree. Costs caused by EF 3 prolong response times for the first task (RT 1). Because the costs occur before the bottleneck, task 2 processing is prolonged by the same amount. In other words, EF 3 defers RT1 and RT2 to exactly the same amount. The effect of EF 2 is hard to assess. On the one hand, according to the traditional locus-of-slack methodology, one would expect EF 2 to be absorbed in the refractory waiting time (termed PRP in Task 2 processing in Fig S2) without

affecting response times. However, on the other hand the demands on EF might in general be more prolonged and distributed across task processing (different to the schematic illustration in Figure S2), and may occur in parallel to other processing stages (Sigman & Dehaene, 2005). For instance, Task 2 inhibition (EF 2) may be required during the whole Task 2 perception stage until the bottleneck is free, and therefore may overlap with the demands to set the bottleneck to Task 1 processing (EF 3). Following the time-based resource-sharing model (TBRS) (Barrouillet, Bernardin, & Camos, 2004), the controlled attention required by EF processes cannot work in parallel, so that a rapid alternating between EF2 and EF3 is expected. As a consequence, EF2 and EF3 are slowed down which then would prolong Task 1 processing before the bottleneck, and consequently also RT1 and RT2. To not rule out this possibility, from now on we refer to costs caused by “EF2 and EF3” with the understanding that this potentially refers only to EF3. Finally, switching of the bottleneck and re-activating of task 2 (EF 4) occurs in task 2 processing only, and only after the bottleneck processing of task 1 has finished. Therefore, costs caused by EF4 prolong only RT2. Taken together, by analyzing the multitasking costs in more detail, in particular by analyzing RT1 and RT2 separately, we can disentangle the relative contributions of the costs appearing before or at the bottleneck (EF 2 and EF 3) from those after the bottleneck (EF 4).

For this, we first analyzed RT1 in the same way as we did with RT2, i.e. we calculated a 2 x 2 factorial ANOVA with the between-subject factor group (experimental vs control) and the within factor task (single-task vs dual-task), but this time using RT1 from the dual-task trials (see analyses in reference to Table 4 in the main manuscript). This analysis is equivalent to what we have done before, and consequently it is again the interaction term of this ANOVA which indicates that the costs are different for the investigated group and the respective control group. This analysis revealed that for all groups the interaction term was statistically significant (all $p < .05$), with the exception of the bilingualism study which only

approached significance (p = .105) (note that the group differences in *costs* presented in Table 4 is equivalent to the interaction term of the ANOVA presented here). The pattern was the same as before, i.e. groups which are known to have impaired executive functions (dyslexics, nicotine deprived, high neurotics) showed larger multitasking costs as their respective controls, while two of the three groups known to have improved executive functions (videogamers, caffeine consumption) showed reduced multitasking costs. Because these costs were calculated by using dual-task RT1 (as opposed to RT2), they cannot be caused by EF4. Therefore, these findings support the conclusion that PRP dual-tasks demand executive functions EF2 and potentially also EF3 (Fig. S2).

The above analysis tested for multitasking costs in RT1. Next, we assessed whether there are any additional multitasking costs in RT2 which go beyond those in RT1 (again, this is a more detailed description of the data in the main manuscript presented in Table 4). Such additional costs should be a good indicator for the costs caused by EF4. Thus, to test for EF 4, we analyzed the difference between RT1 and RT2 (i.e., RT2 – RT1) and tested whether this difference shows significant differences between the experimental and control groups. For this we calculated a 2 x 2 ANOVA with the between-subject factor group (experimental vs control) and the within-subject factor dual-task RT (RT1 vs RT2). As described above, EF 2 and EF 3 have an additive effect on RT2, i.e. if they are the sole cause for the observed overall multitasking costs, RT1 and RT2 should be deferred by the same amount of time, and the interaction term of the ANOVA should not be significant. On the other hand, if EF4 adds a significant amount of deferment to the task processing, RT2 should be deferred more than RT1, and the interaction term should be significant. Because we are testing for a very specific and fine-grained effect for which we have a directed hypothesis, we used one-sided significance testing. Results showed that indeed four of the six groups showed a significant interaction term. In more detail, the dyslexic and neuroticism studies known to have impaired

executive functions showed increased multitasking costs (both p < .05), while the smoking study was not significant (p = .198). The videogaming and bilingual groups, which are known to have improved EF capabilities, showed indeed lower multitasking costs (both p < .05), while the caffeine group only approached significance (p = .108). Therefore, these findings support the conclusion that PRP dual-tasks also demand executive functions EF4 (Fig S2).

The two analyses above complement each other in that their sums constitute the overall multitasking costs. In other words, the costs identified in the main analysis (A: dual-task RT2 – single-task RT1) is the sum of the RT1 costs (B: dual-task RT1 – single-task RT1) and the difference between RT1 and RT2 (C: dual-task RT2 – dual-task RT1): A = B + C.

3. Results – Experiment series 2

3.1. Error rates

In the following, detailed analyses of the error rates for Experiment Series 2 are presented (Fig. S3).

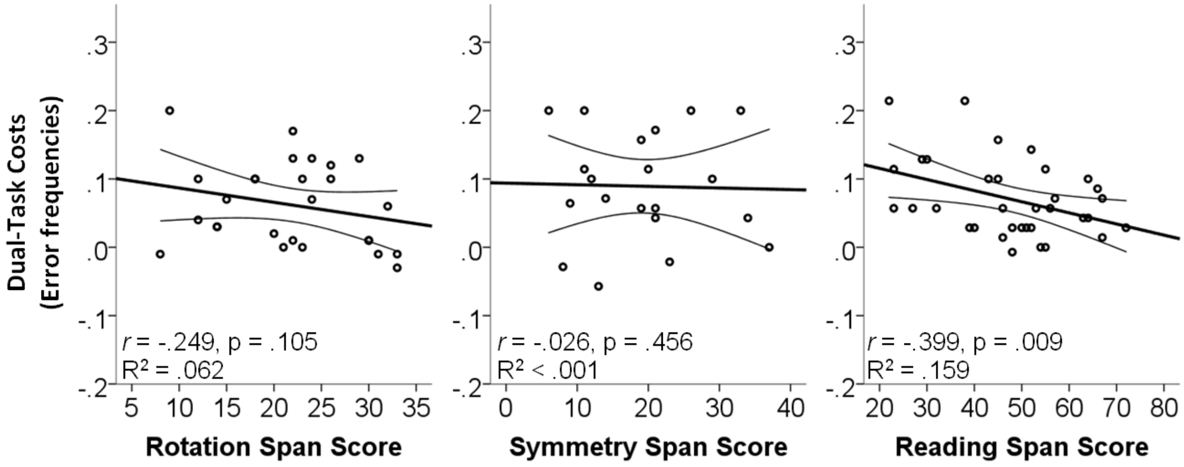

Fig. S3. Scatterplots illustrating the association between dual-task costs and measures of working memory capacity. Dual-task costs (relative error frequencies) were calculated as

dual-task error frequencies minus single-task error frequencies. Each trial was considered only as either correct or erroneous, irrespective of the number of potential errors made in this trial (in dual-task trials, participants could commit more than one error per trial). Span scores are the raw partial scores. Error lines show the SEM of the best line of fit.

3.2.Calculation of (dual-task RT2 – dual-task RT1) in Experiment Series 2

Because running a working memory span task in addition to the PRP task is time consuming, we shortened the PRP task. In more detail, we presented only one dual-task order, i.e. participants always had to respond first to the auditory stimulus and then to the visual stimulus (and never the other way round). Thus, in the dual-task, the first response (RT1) is always from the auditory task, and the second response (RT2) is always from the visual task. Thus, by calculating just the raw difference RT2 – RT1, we would subtract RTs from two different tasks. To alleviate this situation, we first individually adjusted the dual-task response times by the respective single-task response times. Thus, we first subtracted the RTs of the auditory single task from dual-task RT1 (auditory task; $RT1_{\text{-}AUD\text{-}ST}$) and the RTs of the visual single task from dual-task RT2 (visual task; $RT2_{\text{-}VIS\text{-}ST}$). RT2 – RT1 was then calculated based on these relative measures ($RT1_{\text{-}AUD\text{-}ST}$ and $RT2_{\text{-}VIS\text{-}ST}$). However, please note that the raw differences of RT2 – RT1 and the adjusted differences ($RT2_{\text{-}VIS\text{-}ST}$ – ($RT1_{\text{-}AUD\text{-}ST}$) were highly correlated with each other (all $p < .001$), suggesting that the same results would have been observed with the raw differences.

4. SI References

Barrouillet, P., Bernardin, S., & Camos, V. (2004). Time constraints and resource sharing in adults' working memory spans. Journal of Experimental Psychology: General, 133, 83–100.

De Jong, R. (1995). The role of preparation in overlapping-task performance. The Quarterly Journal of Experimental Psychology. A, Human Experimental Psychology, 48, 2–25.

Logan, G. D., & Gordon, R. D. (2001). Executive control of visual attention in dual-task situations. Psychological Review, 108, 393–434.

Luria, R., & Meiran, N. (2003). Online order control in the psychological refractory period paradigm. Journal of Experimental Psychology: Human Perception and Performance, 29, 556–574.

Meyer, D. E., & Kieras, D. E. (1997). A computational theory of executive cognitive processes and multiple-task performance: Part 1. Basic mechanisms. Psychological Review, 104, 3–65.

Monsell, S. (2003). Task switching. Trends in Cognitive Sciences, 7, 134–140.

Sigman, M., & Dehaene, S. (2005). Parsing a cognitive task: a characterization of the mind's bottleneck. PLoS Biol, 3, e37.

Szameitat, A. J., Lepsien, J., von Cramon, D. Y., Sterr, A., & Schubert, T. (2006). Task-order coordination in dual-task performance and the lateral prefrontal cortex: an event-related fMRI study. Psychological Research, 70, 541–552.

Szameitat, A. J., Schubert, T., & Müller, H. J. (2011). How to test for dual-task-specific effects in brain imaging studies--an evaluation of potential analysis methods. NeuroImage, 54, 1765–1773.

Szameitat, A. J., Schubert, T., Müller, K., & von Cramon, D. Y. (2002). Localization of executive functions in dual-task performance with fMRI. Journal of Cognitive Neuroscience, 14, 1184–1199.
